# Reducing sedentary behaviour and physical inactivity in the workplace: protocol for a review of systematic reviews

**DOI:** 10.1136/bmjsem-2020-000909

**Published:** 2020-12-04

**Authors:** Anna Valeria Dieterich, Andre Matthias Müller, Katika Akksilp, Sarin K C, Saudamini Vishwanath Dabak, Thomas Rouyard

**Affiliations:** 1 Saw Swee Hock School of Public Health, National University of Singapore, Singapore; 2 Centre for Sport & Exercise Sciences, University of Malaya, Kuala Lumpur, Malaysia; 3 Health Intervention and Technology Assessment Program, Mueang Nonthaburi, Thailand; 4 Research Center for Health Policy and Economics, Hitotsubashi University, Kunitachi, Japan

**Keywords:** evidence based review, sedentary, sitting time, physical activity

## Abstract

**Background and objective:**

Increasing rates of urbanisation have been accompanied by higher levels of sedentary behaviour (SB) and reduced physical activity (PA) worldwide. While physical inactivity has long been identified as a major risk factor for morbidity and mortality, increased concerns about the detrimental associations between SB and health has led to the development of many interventions aimed at reducing SB and/or promoting PA. Due to the prominence of sedentary time spent at work, the workplace has been identified as a key setting to implement such interventions. Building an evidence base of effective strategies to reduce SB and/or promote PA at work is needed to help reduce the health risks faced by many employees.

**Methods and analysis:**

We aim to conduct a review of reviews (RoR) to identify, evaluate and synthesise all systematic reviews (SRs) of workplace interventions aimed at reducing SB and/or promoting PA among adults. Systematic searches for relevant SRs will be conducted in six databases: Cochrane Systematic Review Database, Cumulative Index to Nursing & Allied Health Literature through EBSCOhost, EMBASE, PubMed including MEDLINE, Scopus and Web of Science. Selection for final inclusion and data extraction will be performed by two independent reviewers. SRs will be included if they assessed interventions aimed at reducing SB or promoting PA in the workplace, and if they report on changes in the respective behavioural outcomes in the occupational domain.

**Discussion:**

This RoR will be valuable to policy-makers and employers who are looking for strategies to promote health at work. This will also allow potential research gaps to be identified, so that the design of future studies can be better informed.

**Trial registeration:**

This study has been registered with the PROSPERO International Prospective Register of Systematic Reviews (registration number CRD42020171774).

**Summary box**

**What is already known?**

- Despite many systematic reviews on interventions aimed at reducing sedentary behaviour (SB) and/or promoting physical activity in the workplace, the diversity of the literature makes it difficult to identify the most promising strategies to date.
- Conducting a ‘review of systematic reviews’ (RoR) is a prime methodological approach to synthesise level 1 evidence on a topic that has generated considerable primary research
- Over the past decades, physical inactivity and SB have been identified as major risk factors for increased mortality and morbidity

**What this work adds?**

- Reporting RoR protocols is essential to ensure the methodological transparency of the work undertaken and to guide the development of future RoRs at a time when this approach is still relatively new

## Introduction

A high proportion of the global population spends too much time being sedentary and not enough time being physically active. According to WHO, approximately 3.2 million deaths each year are attributable to insufficient physical activity (PA).^1^ This trend, which has been increasing over the past decades, largely results from urbanisation, technologisation and the adoption of a ‘Western lifestyle’ across the world. In many high-income countries, sedentary behaviour (SB) amounts to more than half of adults’ waking time (between 8 and 11 hours daily),^2–4^ while 32% of men and 42% of women did not meet the WHO PA guidelines in 2016 (ie, engaging in at least 150 min of moderate intensity PA per week).^5^

Such behavioural patterns have important implications for the health of populations. While the negative effects of physical inactivity have been well documented, recent research suggests that SB (sitting, reclining and lying down) could also act as an independent risk factor for non-communicable diseases and all-cause mortality.^6 7^ If such detrimental effects are confirmed, quantitative SB guidelines will need to be developed alongside existing PA guidelines (despite many public health authorities recommending ‘sitting less’, there is to date no established dose–response relationship to support such guidelines).^8^ Current evidence on the detrimental effects of SB is mainly cross-sectional (and often dependent on PA levels),^9–11^ therefore, caution is required when interpreting the results. Nevertheless, regardless of whether SB is ‘more than a mere absence of PA’ or not, effective behaviour change interventions to reduce SB and promote PA are needed.

In many countries, white-collar work is a major determinant of SB and physical inactivity. A recent study found that office employees in the Netherlands spend around 76%–80% of their working time in a sedentary position,^12^ while 64% of jobs in the USA require being sedentary or, at best, engaging in only minimal PA.^13^ Reflecting the need to promote both the reduction of SB and the substitution of sedentary time by PA, many behaviour change interventions have been implemented in workplaces over the past decade.

The accumulated evidence on the effects of such interventions has been discussed in several systematic reviews (SR).^14–25^ The scope of these reviews largely varies according to whether included primary studies aim at specifically reducing SB,^14–20^ increasing PA^21–24^ or both.^25^ Some SRs have focused on specific types of interventions, such as height-adjustable workstations^16 19^ or cycle and treadmill desks^23^; while others have focused on specific types of outcomes (eg, health outcomes^15 23 24^) or populations (eg, women^24^). In this context, the diversity of the literature makes it difficult to have a clear overview of what works in reducing SB and/or increasing PA at work, for whom, and to which extent. To address this, we aim to conduct a review of reviews (RoR) on the topic. An RoR or ‘overview of reviews’ answers a specific research question by summarising evidence from all available SRs rather than primary studies. Four such RoRs have already been published on SB and/or PA interventions, however, the authors have primarily focused on interventions conducted outside of the workplace, in youth^26 27^ or older populations.^28^ Only one RoR has targeted workplace interventions so far, and it is only available in German language.^29^ A thorough search of titles in the PROSPERO review database confirmed that no such RoR is available or underway.

The objective of our RoR is therefore to identify, evaluate and synthesise all available SRs that investigated the effects of workplace interventions on SB and/or PA. Specifically, we aim to:

- Summarise intervention types, population groups, and settings that have been studied.
- Examine whether and how effects vary according to specific intervention characteristics. We aim to classify interventions according to their target level(s) as defined by the social-ecological model (eg, individual, environmental or multilevel interventions)^30^; and to examine which intervention types are more promising within each level. If possible, we will also assess effects based on intervention goals (eg, merely reducing SB, replacing SB by PA, etc) and measured outcomes (eg, objective or subjective measures).
- Identify research gaps to inform the development of future studies

## Methods

The study protocol was designed in accordance with the Preferred Reporting Items for Systematic Reviews and Meta-Analyses Protocols (PRISMA-P) guidelines.^31^ Despite being a relatively new approach, guidelines on how to conduct and report RoRs have been developed.^32–34^ The recommended methodology largely draws on the PRISMA guidelines developed for SRs of primary studies.^35^ The proposed review will be conducted in accordance with such guidelines. Patients and/or the public were not involved in the design, conduct, reporting or dissemination plans of this research.

### Step 1: identifying relevant SRs

We will use the ‘PICOS’ framework to devise our search strategy. PICOS stands for Population, Intervention, Comparison, Outcome and Study design, and is commonly used to facilitate the identification of components of clinical evidence for SRs.^36^

#### Study design and characteristics

We will include SRs with or without meta-analysis published between January 2000 and April 2020 in peer-reviewed journals or doctoral dissertations. Other types of studies, such as scoping reviews or primary research, will be excluded. Selection of SRs will not be limited by language or geographical location.

#### Population

We will include SRs of studies involving employed adult participants (18 years or older). SR focusing exclusively on employees with limited physical mobility due to a disability, or a health condition will be excluded. SRs in which there is a mixture of participants (ie, only some of them matching these criteria) will also be considered, provided that subgroup analyses are reported.

#### Interventions and settings

Only SRs of workplace interventions addressing the reduction of SB and/or increase of PA in the workplace will be considered for inclusion. Such interventions may include the provision of height-adjustable workstations, exercise classes or general lifestyle interventions. SRs that include both workplace and non-workplace interventions will be included only if they report domain-specific analyses (ie, effects on SB and/or PA during work hours). We will not limit the inclusion of SRs based on the design of primary studies.

#### Outcomes

Only SRs that report on SB and/or PA outcomes during working hours will be considered for inclusion. In SRs where outcomes are reported in various domains, a separate analysis on occupational PA or SB must be provided. PA and/or SB outcomes reported in individual SRs might have been measured objectively (eg, using an accelerometer) or subjectively (eg, through self-reporting or observation).

We will follow the recommendations of the Sedentary Behaviour Research Network^37^ and the guidelines of the American College of Sports Medicine^38^ to define SB and PA outcomes, respectively. SB will be defined as ‘any waking behaviour characterised by an energy expenditure ≤1.5 metabolic equivalents (METs) while in a sitting or reclining posture’. As such, we will include reviews that report on various SB outcomes such as sitting time or breaks from sitting. PA outcomes reported, for their part, can vary widely. We will consider any outcome that indicates activity above the 1.5 METs threshold such as the number of steps, and time spent being active. PA outcomes will be categorised by level of intensity: light PA (≥1.5 METs;≤3 METs), moderate PA (>3 METs;≤6 METs), vigorous PA (>6 METs) if possible.

We will conduct comprehensive searches in the following six electronic databases: Cochrane Systematic Review Database, Cumulative Index to Nursing & Allied Health Literature through EBSCOhost, EMBASE, PubMed including MEDLINE, Scopus and Web of Science. Three themes will be used to identify keywords as part of the search strategy: (1) SB and PA; (2) work environment; (3) SRs (see details in table 1). Published SRs will also be searched to identify additional keywords.^14–25^

**Table 1:** Search strategy (Web of Science)

Groups	Descriptors	Boolean operator	Field searched
SB and PA	inactiv* OR “computer use*” OR sedentar* OR sitting OR reclin* OR stationary OR chair* OR seated OR standing OR lying OR recumben* OR screen* OR “energy expenditure” OR exercise* OR step* OR acceleromet* OR pedomet* OR fitness OR workout OR sport* OR mov* OR walk* OR aerobic* OR yoga OR pilates OR zumba OR treadmill OR cycl* OR stair* OR gym	AND	Ts=title-abstract-keyword search
Study design	“systematic review*” OR “meta-analys*”	AND	Ts=title-abstract-keyword search
Occupational domain	occupation* OR work* OR labo?r OR office* OR job* OR vocation* OR desk* OR “white collar*” OR “white-collar” * OR “call-cent*” OR employe*	AND	Ts=title-abstract-keyword search
Screening	screening	NOT	Ts=title-abstract-keyword search

PA, physical activity; SB, sedentary behaviour.

In addition to these searches, we will search the reference lists of included publications to identify other relevant SRs. Field experts will be also be contacted, and searches on the Sedentary Behaviour Research Network website will be conducted.

### Step 2: selecting relevant SRs

The software Covidence will be used for data management.^39^ Covidence is an SR management software that divides the review process into four stages: import of references, screening by title and abstract, full-text screening and data extraction. Results from all literature searches will be imported into Covidence and duplicates will be removed before the first screening stage.

Selection of relevant SRs will be conducted in two stages. First, two reviewers will independently screen all retrieved records by title and abstract against eligibility criteria. A third reviewer will be consulted to resolve conflicts. Second, full texts of all selected records will be reviewed independently by two reviewers and reasons for exclusion will be recorded. Each reviewer will be trained to ensure screening consistency. The training will consist of each reviewer screening ten full texts. Resulting conflicts will be resolved, and the inclusion and exclusion criteria clarified. Any disagreement at this stage, including any conflicts of reason for exclusion, will be resolved by consulting a third reviewer. If relevant information on eligibility appears to be missing, study authors will be contacted by the review team.

### Step 3: data extraction and synthesis

Two reviewers will be involved in the data extraction stage. The first reviewer will extract all data from included SRs using a previously piloted data extraction form. The second reviewer will check all the extracted data for accuracy. In case of disagreements, a third reviewer will be consulted or study authors will be contacted for clarification.

The following data items will be extracted from included SRs:

- Author(s), title, type of publication, date of publication, funding source.
- Aim(s), design (with or without meta-analysis).
- Search strategies (eg, databases searched, dates searched, etc).
- Main characteristics of included primary studies (eg, design, population, etc).
- Intervention types examined.
- Outcomes reported in the form of a narrative synthesis or a meta-analysis, including subgroup analyses.
- Risk of bias assessment of included studies.
- Recommendations (policy and future research).

We will summarise the evidence on the effects of interventions on SB and/or PA using a narrative synthesis approach. The narrative synthesis will be structured by intervention level (individual, physical environment, social environment and multicomponent interventions), and subsequently by intervention type within each level category. In order to make the elicitation of the main findings easier for the reader, we will also present the results in tabulated form. For each type, we will report the number of reviews and primary studies included, the type of outcome(s) measured, as well as a summary of the available evidence (effects and bias assessed by AMSTAR 2), where possible. Finally, if data permit, we will report findings related to process outcomes such as adherence, acceptability and satisfaction associated with each type of intervention.

### Step 4: assessing risk of bias

Two reviewers will appraise the risk of bias in each included SR using A MeaSurement Tool to Assess systematic Reviews (AMSTAR) 2 instrument.^40^ The first reviewer will appraise the risk of bias of each included SR while the second reviewer will check all appraisals. A third reviewer will be consulted in case of disagreements. Several dimensions will be assessed, including potential publication bias, conflicts of interest and appropriateness of the statistical methods used if the authors conducted a meta-analysis. The AMSTAR 2 criteria will guide the classification of SRs into four categories indicating the confidence in findings: high, moderate, low, critically low. The cumulative evidence reported in the review will be interpreted in this light. Recommendations for policy-makers and employers, as well as for future research, will be drawn accordingly.

## Discussion

Physical inactivity, and more recently SB, have been identified as major risk factors for increased mortality and morbidity. Reflecting an increased concern in many countries, a vast number of interventions have been implemented to reduce SB or promote PA among sedentary workers. Despite several SRs published on the topic, it is still unclear how effective various intervention types are in changing behavioural outcomes. This RoR aims to fill this gap. Conducting an evidence-based synthesis will be valuable to both policymakers and employers who are looking for effective and scalable strategies to promote health at the workplace. In particular, there is an increasing demand for such strategies from governments and private parties in low-income and middle-income countries, where predominantly rural economies are rapidly shifting towards more urban ones. For example, in Thailand, the national Health Promotion Foundation has recently commissioned the ‘Physical Activity at Work’ research programme to help address the increasing rates of SB in the population.^41^ Our findings will also further inform the implementation of workplace interventions in countries where guidelines or nationwide initiatives to reduce SB or increase PA already exist, such as Australia,^42^ UK^43^ or Singapore.^44^ Finally, this RoR will also allow potential research gaps to be identified, so that the design of future studies can be better informed.
